# Clinical Characteristics and Outcomes of Acute Lymphoblastic Leukemia in Adolescents and Young Adults in Malawi

**DOI:** 10.1200/GO.21.00388

**Published:** 2022-06-30

**Authors:** Edwards Kasonkanji, Stephen Kimani, Brent Skiver, Grace Ellis, Ryan Seguin, Bongani Kaimila, Tamiwe Tomoka, Maurice Mulenga, Nathan Montgomery, Yuri Fedoriw, Satish Gopal, Katherine D. Westmorland, Matthew S. Painschab

**Affiliations:** ^1^ University of North Carolina Project-Malawi, Lilongwe, Malawi; ^2^ Huntsman Cancer Institute, University of Utah, Salt Lake City, UT; ^3^ Messino Cancer Center, Asheville, NC; ^4^ Lineberger Comprehensive Cancer, University of North Carolina, Chapel Hill, NC; ^5^ University of Malawi College of Medicine, Blantyre, Malawi; ^6^ Malawi Ministry of Health, Lilongwe, Malawi; ^7^ Center for Global Health, National Cancer Institute, Rockville, MD

## Abstract

There are limited data on treatment and outcomes for acute lymphoblastic leukemia (ALL) among adolescents and young adults in sub-Saharan Africa. We describe a prospective observational cohort in Malawi.

**METHODS:**

Patients age 15-39 years with newly diagnosed ALL at Kamuzu Central Hospital, Malawi, were enrolled from 2013 to 2019; follow-up was censored on December 2020. ALL diagnosis was confirmed on-site using immunohistochemistry and telepathology consultation involving pathologists in Malawi and the United States. All but four patients were treated with a modified pediatric-inspired regimen (Cancer and Leukemia Group B 10403 protocol). Key modifications included omission of asparaginase and no dose escalation for methotrexate.

**RESULTS:**

Of 19 participants, the median age was 22 (range 15-36) years. Of the 15 patients who initiated treatment, 11 (73%) achieved remission after induction, one (7%) died during induction, two (13%) had refractory disease, and one (7%) absconded. No patients were lost to follow-up. Eventually, 10 of 11 patients (91%) with confirmed remission relapsed. The median duration of first remission was 10 (range 3-22) months. Twelve of 15 treated patients (80%) had died at the time of censoring. Among treated patients, the 12- and 24-month overall survival was 50% (95% CI, 23 to 72) and 17% (95% CI, 3 to 42), respectively. CNS involvement was associated with worse survival.

**CONCLUSION:**

It is possible to treat adolescents and young adults with ALL in low-resource settings using a low-cost, pediatric-inspired regimen; however, outcomes are poor. Both cost and limitations in supportive care infrastructure limit intensive cytotoxic approaches such as asparaginase. Patient-reported outcomes are needed to understand the quality of life and cost-effectiveness. Critically, innovative, leap-frog therapies, such as monoclonal or bispecific antibodies, and feasible economic models for resource-limited settings are urgently needed.

## INTRODUCTION

Acute lymphoblastic leukemia (ALL) is associated with a bimodal age distribution, with one peak in early childhood (median age 8-9 years) and one between the fourth and fifth decades of life.^1^ Prognosis is influenced by age and genetic characteristics.^2^ Although childhood ALL in high-income countries has very high cure rates of around 90%,^2^ outcomes among adults remain poor, with < 45% of affected patients expected to achieve long-term disease-free survival.^1,3^ Advancements in intensive therapeutic regimens and the preponderance of favorable genetic characteristics in the pediatric population partly drive the difference in survival outcomes between childhood and adult ALL.^1^

**CONTEXT**

**Key Objective**
Acute lymphoblastic leukemia (ALL) is an aggressive leukemia that is rapidly fatal if untreated. The prognosis for adolescents and young adults (AYAs) in high-income countries has improved significantly with the use of pediatric-inspired regimens. However, little is known about ALL outcomes in resource-limited settings where both supportive care infrastructure and access to newer, less toxic therapies are limited.
**Knowledge Generated**
This study provides one of the first descriptions of ALL among AYAs treated with a pediatric-inspired regimen in a low-resource setting. We identified 19 AYAs with ALL from 2013 to 2019 in Malawi. Of the 15 patients who initiated treatment, 11 (73%) achieved remission after induction, one (7%) died during induction, two (13%) had refractory disease, and one (7%) absconded. Among treated patients, the 12- and 24-month overall survival was 50% and 17%, respectively.
**Relevance**
Improvements in both implementation of currently available therapies and access to innovative therapies are urgently needed to reverse this cancer inequity.

Given poor outcomes of ALL among adults, multiple strategies to improve outcomes have been explored. One key observation is that adolescents and young adults (AYAs; defined as age 15-39 years) with ALL treated on pediatric-inspired regimens have better outcomes, including survival, hospital time, toxicities, late effects, and quality of life during and after treatment compared with adult oncology regimens.^4-6^ In the recently published Cancer and Leukemia Group B (CALGB) 10403 study, after 3 years, 73% of participants had achieved and remained in remission.^7^ Unfortunately, descriptions of treatment and outcomes of nonpediatric ALL from low- and middle-income countries, where treatment is challenging because of limited supportive care infrastructure, are scarce.^8^ Additional challenges include lack of access to frontline drugs such as asparaginase, poor diagnostic infrastructure, high treatment default rates, and toxicity-related complications.^9^ This study provides one of the first descriptions of treatment and outcomes of ALL among AYAs treated with a pediatric-inspired regimen in a low-resource setting.

## METHODS

We identified newly diagnosed, pathologically confirmed ALL in patients age 15-39 years enrolled in an observational, prospective cohort at Kamuzu Central Hospital from June 2013 to December 2019. Kamuzu Central Hospital is a referral hospital located in Lilongwe, the capital city of Malawi, that provides cancer diagnostic and treatment services to a catchment area of approximately nine million people.

All patients underwent a comprehensive baseline evaluation, including chest x-ray, abdominal ultrasound, CSF cytology, diagnostic bone marrow evaluation, and excisional lymph node biopsy (if applicable). In addition, immunophenotyping was performed on-site in Malawi using immunohistochemical stains for CD3 (clone PS1), CD20 (clone L26), CD30 (clone 15B3), CD45 (code NCL-L-LCA-RP), CD138 (clone MI15), BCL2 (clone bcl-2/100/D5), Ki-67 (clone MM1), and TdT (clone TdT-338) from Leica Biosystems (Buffalo Grove, IL). Pathology assessments were supported by real-time telepathology consultations involving two to four pathologists in Malawi and the United States. The US-based pathologists participated in weekly consensus telepathology conferences with the Malawi-based colleagues and confirmed cases subsequently underwent a secondary review of cases in Chapel Hill as previously described.^10^

Patients were treated with a modified regimen modeled after the CALGB10403 protocol for AYAs.^7^ Treatment phases included induction, consolidation, interim maintenance, delayed intensification, and maintenance (Table 1). However, because of resource limitations within the public health system, we omitted asparaginase from the regimen, and we did not initially escalate methotrexate during interim maintenance because of the inability to measure methotrexate levels and possible differences in methotrexate clearance in different populations. The latter has subsequently been implemented without complications. Postinduction bone marrow biopsy was used to assess remission status. All patients received standardized anti-infective prophylaxis with ciprofloxacin, fluconazole, and cotrimoxazole during induction until neutrophil count recovery (ie, absolute neutrophil count ≥ 500 × 10^9^ cells/L), with the continuation of cotrimoxazole and acyclovir throughout the treatment courses.

**TABLE 1:**
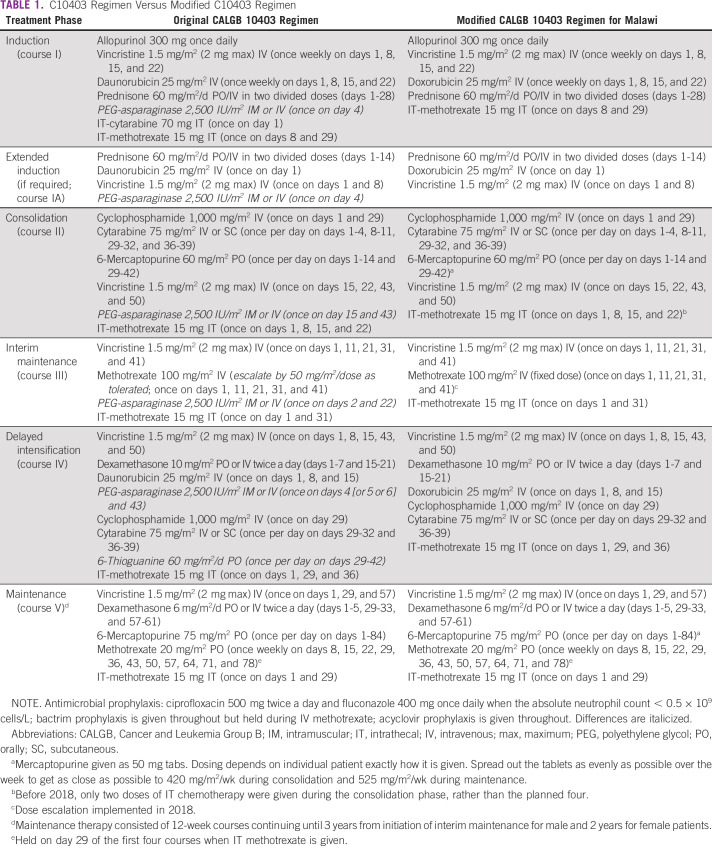
C10403 Regimen Versus Modified C10403 Regimen

We actively followed patients until death or administrative censoring on December 31, 2020. Possible outcomes included alive, dead, lost to follow-up (defined as no form of contact after > 3 phone calls and physical tracing), and absconded (defined as abandoning treatment but still able to contact the participant).

We used descriptive statistics to assess patient characteristics and overall survival (OS) using Kaplan-Meier methods. Univariate analysis of predictors of OS were compared by the log-rank test. We used Stata version 13 (College Station, TX) and R (Vienna, Austria) to analyze data; figures were produced using the R package ggplot2.^11^ This study was approved by the Malawi National Health Science Research Committee and the Institutional Review Board of the University of North Carolina at Chapel Hill. All patients provided written informed consent.

## RESULTS

### Baseline Characteristics

From June 2013 to December 2019, we identified 19 AYAs with newly diagnosed ALL (Table 2). Thirteen patients (68%) were male, and the median age was 19 (range 15-36) years. All patients were HIV-negative. At diagnosis, 10 patients (53%) endorsed symptoms for 3 months or fewer; three had a history of tuberculosis treatment, including two for whom tuberculosis treatment was prescribed for adenopathy related to their leukemia. Twelve (63%) had an Eastern Cooperative Oncology Group performance status of ≥ 2. Six (32%) patients had CNS involvement at the time of presentation. Eleven (58%) patients had T-cell ALL by immunohistochemistry. Six (32%) patients presented with leukocytosis > 50 × 10^9^/L, including three with hyperleukocytosis (WBC > 100 × 10^9^/L).

**TABLE 2:**
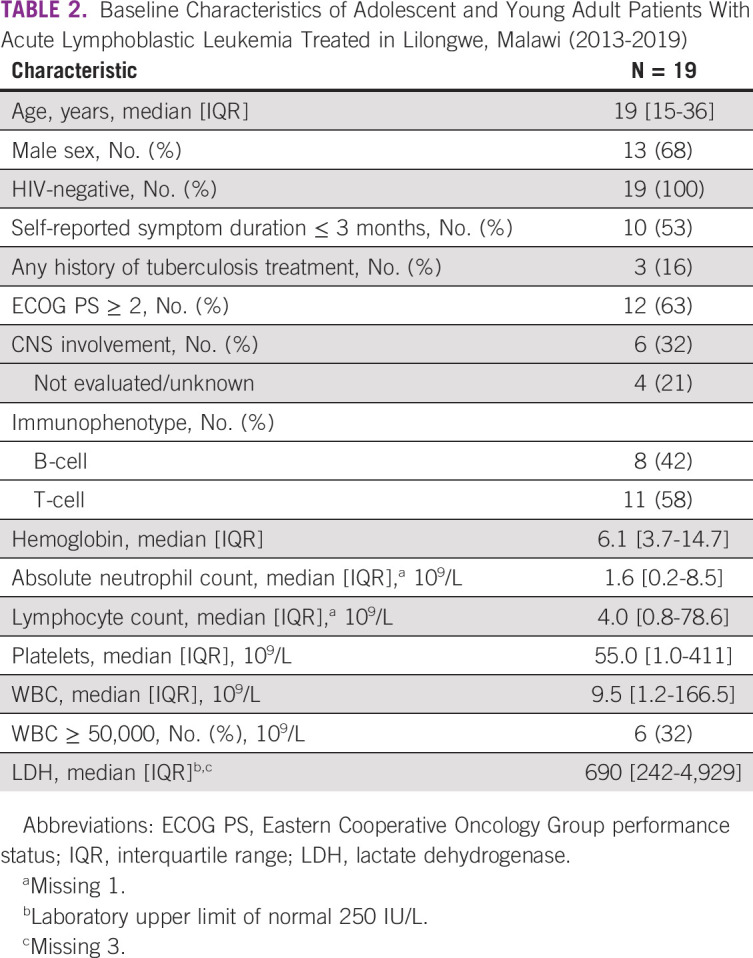
Baseline Characteristics of Adolescent and Young Adult Patients With Acute Lymphoblastic Leukemia Treated in Lilongwe, Malawi (2013-2019)

### Treatment Characteristics and Toxicity

Fifteen patients started therapy, and because of relapse or death, 11 (73%) entered consolidation, eight entered interim maintenance, seven entered delayed intensification, and five entered maintenance (Table 3). Toxicity varied by treatment course; during the course of therapy, nine (60%) participants experienced grade 3 or 4 neutropenia, and most commonly during induction, five (33%) experienced grade 3 or 4 anemia and six (40%) experienced grade 3 or 4 thrombocytopenia. Nonhematologic grade 3 or 4 toxicities that occurred on greater than one occasion included vomiting, diarrhea, epistaxis, constipation, and decreased vision.

**TABLE 3:**
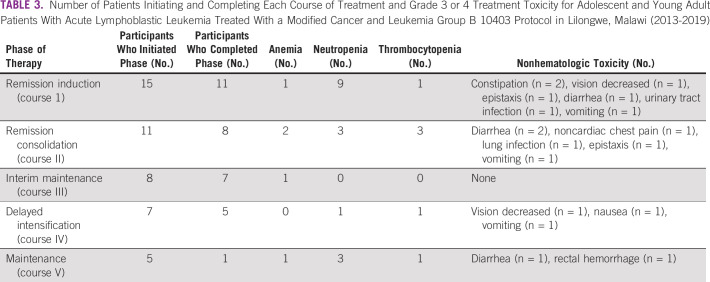
Number of Patients Initiating and Completing Each Course of Treatment and Grade 3 or 4 Treatment Toxicity for Adolescent and Young Adult Patients With Acute Lymphoblastic Leukemia Treated With a Modified Cancer and Leukemia Group B 10403 Protocol in Lilongwe, Malawi (2013-2019)

### Outcomes

**FIG 1:**
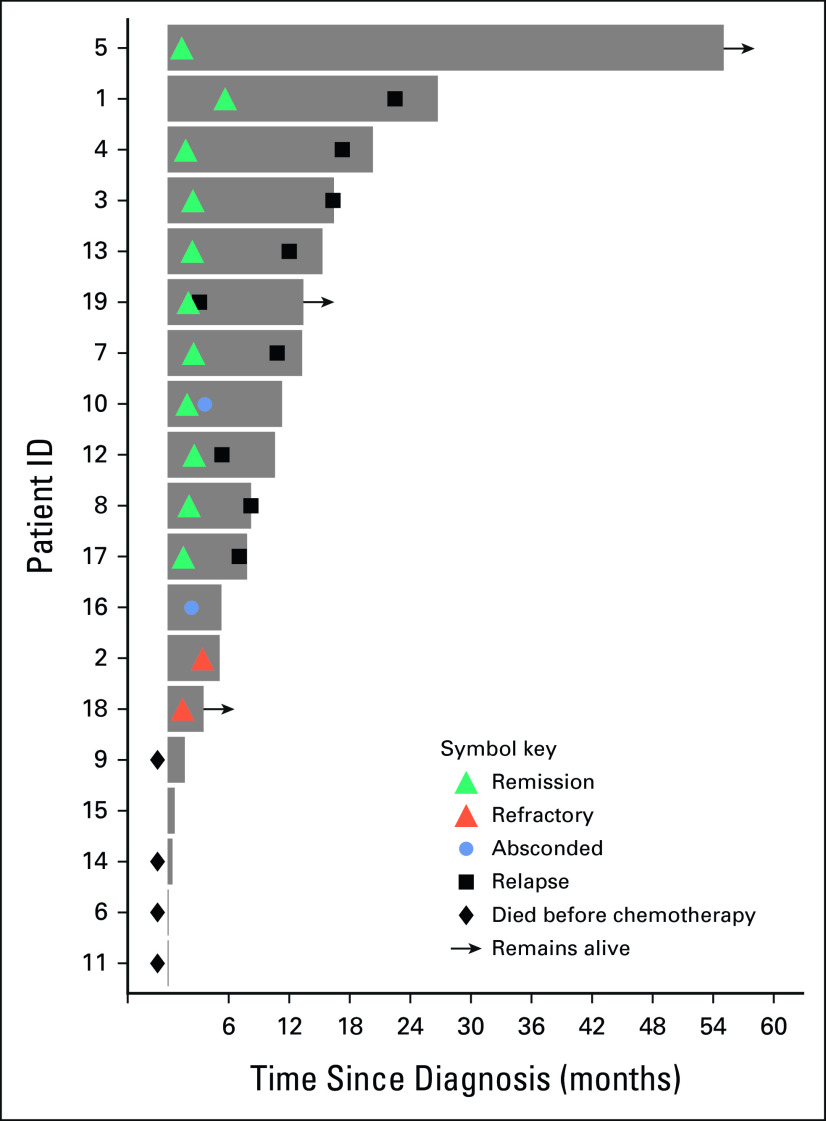
Treatment outcomes for adolescents and young adults with acute lymphoblastic leukemia treated in Lilongwe, Malawi (2013-2019), shown as a swimmer plot.

**FIG 2:**
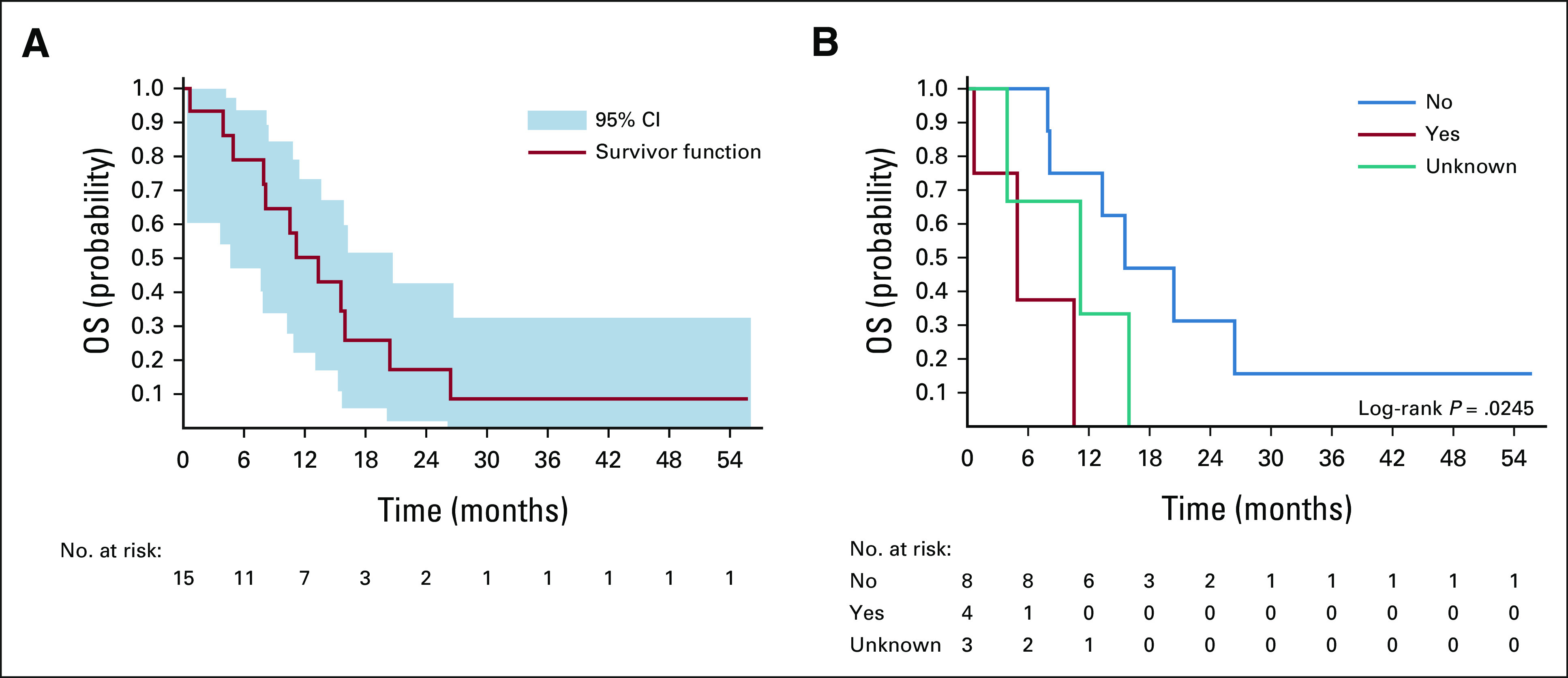
OS for adolescent and young adult patients with ALL (A) among all patients who initiated treatment (n = 15) in Lilongwe, Malawi (2013-2019) and (B) by CNS involvement at baseline. No: not involved; yes: involved; unknown: no clinical signs of CNS involvement but CNS not evaluated before treatment initiation. ALL, acute lymphoblastic leukemia; OS, overall survival.

As of December 31, 2020, no patients were lost to follow-up. The median time from screening to chemotherapy initiation was 7 (range 0-44) days, during which patients could receive prephase corticosteroids. Four patients died before initiating chemotherapy (Fig 1). Of 15 patients who initiated treatment, 11 (73%) achieved remission after induction, one (7%) died during induction, two (13%) had refractory disease, and one (7%) absconded without achieving remission. Subsequently, 10 of 11 (91%) who initially achieved remission relapsed, including one patient who absconded shortly after achieving remission. The median duration of remission was 10 (range 3-22) months. All remissions were bone marrow remissions with peripheral blasts and cytopenias; there were no documented cases of CNS relapse. As of December 31, 2020, three patients were alive: one remained in remission, off maintenance chemotherapy, and without evidence of progression at 56 months, one was in remission in the maintenance phase, and one was censored after being refractory to induction and transferring care to the pediatric center. Of the 15 patients who began chemotherapy, 12 (80%) died, and only one (7%) death was possibly treatment-related. Among all patients, event-free survival, defined as relapse, and loss to follow-up, treatment abandonment, or death at 12 and 24 months were 26% (95% CI, 12 to 56) and 5% (95% CI, 1 to 36), respectively. Among treated patients, the event-free survival at 12 and 24 months was 33% (95% CI, 16 to 68) and 7% (95% CI, 1 to 44), respectively. The OS at 12 and 24 months (N = 19) was 40% (95% CI, 18 to 61) and 14% (95% CI, 2 to 34), respectively. Among treated patients (n = 15), the 12- and 24-month OS was 50% (95% CI, 23 to 72) and 17% (95% CI, 3 to 42), respectively (Fig 2A). In univariate Kaplan-Meier analysis comparing baseline characteristics, only baseline CNS involvement was associated with worse survival (*P* = .02; Fig 2B).

## DISCUSSION

To our knowledge, this is the first description of treatment and outcomes of ALL among AYAs in sub-Saharan Africa. We enrolled participants with advanced disease characteristics (63% Eastern Cooperative Oncology Group ≥ 2, 84% extramedullary involvement, and 32% white cell count > 50 × 10^9^ cells/L). Our study provides one of the most well-characterized cohorts of ALL among AYAs in the region. No patients were lost to follow-up. We initiated treatment at a median of 7 days after the initial screening visit, and we were able to maintain patients in treatment with minimal treatment abandonment, a common confounding factor in the pediatric hematologic malignancy literature from the region.^12-14^

Survival and cure rates with the regimen used in this study were lower than desired. The use of a modified pediatric regimen to treat ALL among AYAs in Malawi resulted in the 12- and 24-month OS of 50% and 17%. Although this is expectedly poor compared with outcomes for AYAs in high-resource settings (3-year progression free survival of 59% in CALGB10403),^7^ it is similar to published outcomes among pediatric ALL from the region.^12,15,16^ For reference, the 2-year OS rate for a predominantly pediatric population receiving an asparaginase-based pediatric regimen for ALL in Rwanda was 22%.^12^ Unsurprisingly, in our study, poor outcomes were related to ALL progression and NOT toxicity, similar to published data for ALL among children in Malawi and Rwanda.^12,16^

Studies from high-income settings suggest that including asparaginase in pediatric-inspired protocols for AYAs with ALL is critically important. In large cohorts of pediatric and AYA ALL patients from high-income countries, discontinuation of asparaginase at any point during therapy was associated with a 5%-10% increase in absolute risk of relapse.^17,18^ The increased risk of relapse may be due to other factors correlated with discontinuation of asparaginase, but certainly the ability to implement complete, maximum-intensity courses of ALL therapy is critically important for survival of patients with ALL. Because of cost and difficulties with implementation, such as management of hypersensitivity and adverse events, asparaginase is not available in the Malawi public health sector and was therefore not included in the regimen reported in this article.^6,19^ Furthermore, compared with children, asparaginase is more challenging to administer in adults because of increased adverse events.^19,20^ Therefore, the effect on survival in sub-Saharan Africa should be explored systematically and, perhaps, adult regimens without asparaginase^21^ or leap-frog regimens including monoclonal antibody therapies^22,23^ or bispecific antibodies^24^ will be more effective in resource-limited settings if equitable price models can be arranged.

CNS involvement at baseline was quite common and predicted poor outcomes in this study. The incidence of CNS disease at presentation in our study is much higher than that seen in high-income countries where it is typically < 10%.^25^ This may be due to chance (ie, small sample size), late presentation of disease, or differences in biology although additional studies are needed to further explore this. In addition, CNS involvement at baseline was associated with increased mortality in our study. This is in line with data from high-income countries where CNS involvement is a poor prognostic factor in adult ALL. Multiple factors might have contributed to this outcome in our population; some have been acted on, and others are room for improvement. Critically, any of the treatments that are typically given for CNS3 disease are not available in the case of cranial irradiation, not routinely given because of risk of high toxicity with the inability to monitor levels in the case of high-dose intravenous methotrexate, or difficult to implement consistently in the case of weekly or twice weekly intrathecal chemotherapy until blast clearance. Each of these are potential areas to target for improvement of outcomes in this population.

Furthermore, in addition to asparaginase availability and treatment of CNS disease, a number of other factors might have contributed to the poor survival outcomes in this study, including, among others, lack of oncology specialists, social determinants of health including poverty and distance from care in a largely rural population, and physical and human resources that may affect prevention and management of toxicities. In addition, survival for some patients in this age group could have been improved with access to Philadelphia chromosome testing tyrosine kinase inhibitor therapy; however, testing and therapy for Philadelphia chromosome have only recently become available. Context-appropriate implementation studies measuring, evaluating, and/or addressing possible contributing factors are urgently needed to assess the effects on outcomes and toxicity.

Our study included a small sample of patients treated at a single tertiary care center in Malawi, and its findings may not be generalized. However, given the lack of similar data from sub-Saharan Africa, our results could inform emerging cancer treatment programs and priorities in the region. Our experience reflects several noteworthy achievements for a difficult-to-treat population in a low-resource setting. First, we robustly identified and characterized ALL, including immunophenotyping. Second, we implemented a contemporary protocol under local conditions adapted from current high-income country practice, which led to the induction of histologically confirmed remission in most patients. Third, we had a minimal loss to follow-up despite a long treatment course. Finally, despite eventual disease progression and manageable toxicity, treatment was delivered on a largely outpatient basis, and patients were able to spend time with their families. Although not formally assessed in this study, we plan to incorporate the use of patient-reported instruments to capture quality of life and symptom burden in this population.

In conclusion, our experience demonstrates that it is possible to deliver a complex chemotherapy regimen in a highly resource-constrained setting although limitations in treatment intensity because of supportive care limitations and asparaginase availability contributed to poor OS for what is a highly curable disease in high-income countries. Context-specific refinement of ALL treatment in resource-limited settings to better approximate curative standards of care in high-income countries will likely lead to improved outcomes. Stakeholders have an ethical imperative to achieve equitable access to curative therapies regardless of the setting.
